# Validity and Reliability of Wearable Motion Sensors for Clinical Assessment of Shoulder Function in Brachial Plexus Birth Injury

**DOI:** 10.3390/s22239557

**Published:** 2022-12-06

**Authors:** Helena Grip, Anna Källströmer, Fredrik Öhberg

**Affiliations:** 1 Department of Biomedical Engineering, Radiation Sciences, Umeå University, 901 87 Umeå, Sweden; 2 Department of Surgical and Perioperative Sciences, Umeå University, 901 87 Umeå, Sweden

**Keywords:** inertial movement unit, brachial plexus birth injury, clinical evaluation, kinematic analysis, shoulder function, scapula movement

## Abstract

The modified Mallet scale (MMS) is commonly used to grade shoulder function in brachial plexus birth injury (BPBI) but has limited sensitivity and cannot grade scapulothoracic and glenohumeral mobility. This study aims to evaluate if the addition of a wearable inertial movement unit (IMU) system could improve clinical assessment based on MMS. The system validity was analyzed with simultaneous measurements with the IMU system and an optical camera system in three asymptomatic individuals. Test–retest and interrater reliability were analyzed in nine asymptomatic individuals and six BPBI patients. IMUs were placed on the upper arm, forearm, scapula, and thorax. Peak angles, range of motion, and average joint angular speed in the shoulder, scapulothoracic, glenohumeral, and elbow joints were analyzed during mobility assessments and MMS tasks. In the validity tests, clusters of reflective markers were placed on the sensors. The validity was high with an error standard deviation below 3.6°. Intraclass correlation coefficients showed that 90.3% of the 69 outcome scores showed good-to-excellent test–retest reliability, and 41% of the scores gave significant differences between BPBI patients and controls with good-to-excellent test–retest reliability. The interrater reliability was moderate to excellent, implying that standardization is important if the patient is followed-up longitudinally.

## 1. Introduction

Brachial plexus birth injury (BPBI) is the most common peripheral nerve injury in children, and it affects motor and sensory functions in the upper extremity in 1.74 per 1000 live births [1]. Complications related to BPBI are scapular dyskinesia, muscle contractures, muscle weakness, joint deformity, and, the most common, internal rotation of the shoulder and flexion of the elbow, depending on the severity and level of injury [2]. There is currently no consensus on how to determine which patients will recover completely [3], and the choice of treatment strategy, timing of rehabilitation efforts, and outcomes after interventions remain a clinical challenge [4,5]. The use of CT (computed tomography), MRI (magnetic resonance imaging), and nerve conduction studies can aid in determining the extent of injury [6]. The time from injury to the onset of functional recovery and its magnitude give important clues to determine the location and severity of the injury and adequate treatment, such as surgical interventions (e.g., nerve grafting, nerve transfers, the release of contractures, tendon–muscle transfers, and injection of botulinum toxin A) [7,8]. Physiotherapy and occupational therapy play an important role in habilitation and postsurgery rehabilitation with a focus on maintaining and improving range of motion, muscle strength, endurance, coordination, motor re-education, and participation in age-appropriate activities [9]. Range of motion and muscle function need to be monitored throughout childhood and adolescence since complications can occur at different ages. Hence, systematic, objective assessment is crucial to determine which interventions are appropriate and for evaluating the outcome of those interventions.

Visual grading based on the Active Movement Scale [10] and modified Mallet score [11] was developed to facilitate long-term follow-ups for range of motion and muscle function in BPBI patients and is used extensively throughout the world [5]. The scales are efficient and easy to implement in clinic but have been criticized as too subjective and missing crucial functional deficiencies [12,13,14,15,16]. Further, these scales do not describe how the glenohumeral joint and scapulothoracic articulation contribute to global shoulder (i.e., thoracohumeral) shoulder motion. The coordinated movement in the glenohumeral and scapulothoracic joints, referred to as the scapulohumeral rhythm, is indeed important for the optimal function of the shoulder [17,18,19]. Goniometry and Medical Research Council 0–5 grading [20] are alternative methods to assess changes in range of motion and muscle strength but are difficult to implement in infants and younger children. Another approach is to use a three-dimensional kinematic analysis of the shoulder complex [12,21,22,23]. Such methods have been used to identify deficient external rotation in the glenohumeral joint and the global movement of the shoulder in children with BPBI when performing tasks of the modified Mallet scale [12,24]. Further, a recent study evaluated a clinical tool that incorporates real-time visual feedback with three-dimensional optical motion capture to measure and analyze shoulder function in BPBI patients [25]. Optical motion capture, a gold standard technique, requires special movement laboratories [26], which makes it unsuitable for regular assessments of shoulder function. Another study used inertial movement unit (IMU) sensors on the thorax and arms with a machine learning algorithm to classify BPBI patients and controls based on arm and trunk movement [27]. As recently reviewed, wearable inertial movement units (IMUs) based on accelerometers, gyroscopes, and/or magnetometers have the potential to be used in clinical assessments, but there is a need for reliable and standardized measurement and analysis methods to achieve sufficient accuracy and give robust clinical applications [28]. This is especially important when analyzing shoulder complexes since scapular movements are small and associated with disturbances from skin and tissue movement.

In this study, we evaluated whether kinematic measures from a wearable IMU system are valid and reliable for clinical assessment of shoulder, glenohumeral, and scapulothoracic function after brachial plexus injury. The validity was analyzed with simultaneous measurements with an optical motion capture system, and the test–retest and interrater reliability were analyzed in a group of six BPBI patients and a group of nine non-asymptomatic controls.

## 2. Materials and Methods

### 2.1. Participants

This study consisted of two parts. Part I analyzed system validity based on comparisons between the wearable IMU system and a gold-standard optical 3D system. For this analysis, three healthy adults with no known shoulder pathology were recruited from the hospital staff (age 40 ± 9, BMI 23 ± 1). Part II analyzed the test–retest reliability and interrater reliability of the IMU system. For these analyses, two groups were recruited, see Table 1. The BPBI group consisted of six children and young adults diagnosed with BPBI, aged between 8–22 years, recruited from patients referred to the Hand and Plastic Surgery Clinic at the University Hospital of Umeå, Sweden. Criteria for inclusion were diagnosis of BPBI and exclusion criterion was not being able to follow simple instructions. The control group consisted of nine age-matched persons, aged 7–25 years, recruited from the Umeå University medical program and family members and acquaintances of hospital staff. A double-sided *t*-test confirmed that age did not differ between groups (*p* = 0.38). Exclusion criterion in this group was known as shoulder pathology. All participants signed informed consent before being included in the study. The study was performed according to the Declaration of Helsinki and was approved by the Regional Ethics committee of Umeå, Sweden (protocol code: 2018/236-31).

### 2.2. Test Procedure

The clinical test battery followed the clinical procedure used for BPBI patients at the Hand and Plastic Surgery Unit, University Hospital of Umeå, Umeå, Sweden. The battery consisted of three general assessments of joint mobility (shoulder flexion–extension, elbow flexion–extension, and forearm pronation–supination) followed by six tasks from the modified Mallet scale (MMS1-MMS6) assessing grade of the plexus injury [11]:

1. Shoulder flexion–extension: The participant lifted both arms as high as possible in the sagittal plane, then back to neutral position. Arms were then extended maximally backward and then returned to neutral.
2. Elbow flexion–extension: The participant lifted both arms as high as possible in the sagittal plane, then back to neutral position. Arms were then extended maximally backward and then returned to neutral.
3. Forearm pronation–supination: The participant held both arms straight with palms facing anteriorly if possible and then flexed both elbows to the horizontal plane (~90 degrees). Maximal forearm pronation and supination were performed by rotating the forearms inwards and outwards.
4. MMS1 global abduction: Maximal abduction was performed by lifting both arms as far as possible in the frontal plane with straight arms and then returning to neutral position.
5. MMS2 global external rotation: The participant first flexed the elbows approximately 90 degrees and was then instructed to move forearms inwards as much as possible before performing the maximal outward rotation of the shoulders by moving forearms outwards as far as possible.
6. MMS3 hand to neck: The participant lifted one hand at a time and placed it on the back of the neck before returning to neutral position. The participant was encouraged to perform the movement with control if high speed was used to reach the neck.
7. MMS4 hand to spine: The participant lifted one hand at a time and placed the back of the hand on the back, reaching as high up as possible. The participant was encouraged to perform the movement with control if high speed was used to reach the back.
8. MMS5 hand to mouth: The participant lifted one hand at a time to the mouth, placing the fingertips over the mouth.
9. MMS6 internal rotation. The participant put the palm of the hand, one at a time, over the navel.

The tasks were standardized so that the participant sat on a wooden chair with hips, knees, and ankles at 90°, feet placed at hip distance apart, and back held upright. Each task began with the participant sitting as still as possible in a neutral position. The neutral position was defined as holding the arms alongside the body with palms facing the body. This position could not be achieved by all participants in the BPBI group, who then tried to achieve this position as best they could. All tasks were performed at self-selected speed, repeated five times, and started and ended in the neutral position. The test leader stood in front of the participant and performed the task together with the participant. See Supplementary Figure S1 for further description of these movements.

The measurements in Part I (validity) took place in a clinical movement analysis laboratory (U-Motion Laboratory, Umeå University, Sweden). The test leader guided the participants through the clinical test battery while movement registration was simultaneously recorded with the IMU system and the optical camera system.

The measurements in Part II (reliability) were carried out at the Hand and Plastic Surgery Unit at Umeå University Hospital (Umeå, Sweden). Two test leaders participated, a physiotherapist, and a medical student. All participants in Part II attended on two occasions within an average of 4.8 days (Table 1). They were instructed to avoid heavy physical activity of the upper extremities two days prior to each occasion. On each occasion, the clinical test battery was carried out by one of the test leaders. The BPBI patients did an extra session on the first occasion performed by the second test leader. Prior to this extra session, the first test leader removed all IMUs from the patient. The patient then had a short rest before the second test leader entered and mounted all equipment again and performed the extra session. One of the test leaders did a full test session while the other test leader did a subset of the test session, including hand to neck and global external rotation, to avoid fatigue. The order between the full session and the short session was randomized.

### 2.3. Equipment and Sensor Placement

An optical camera system with eight 3D cameras and reflective surface markers (Oqus, Qualisys AB, Gothenburg, Sweden) was used as the gold standard system in Part I. The wearable motion analysis system (MoLab^TM^, AnyMo AB, Umeå, Sweden) used in Part I and Part II consisted of six lightweight IMU sensors with 3D gyroscopes (16-bit, range ±2000°/s), 3D accelerometers (16-bit, range ±16 g), and 3D magnetometers (13-bit, range ±1200 μT). The system had been validated for shoulder and elbow motion in previous studies with high within-subject reliability for selected outcome variables and high accuracy in angular data compared to optical motion capture systems and had a systematic error of a few degrees [29,30]. The magnetometers were calibrated prior to each test occasion according to the system’s described calibration routine [31]. The sampling frequency was set to 100 Hz, and data were sent wirelessly to a laptop (Dell Latitude 7400, Intel i5, 8 GB ram). The room was controlled for magnetic disturbances by slowly moving a compass in the measurement area. If the compass needle did not change direction during this examination, the area was assumed to be free of magnetic disturbances. The equipment close to the measurement place was checked to ensure that no metallic details would cause magnetic disturbances.

**Figure 1:**
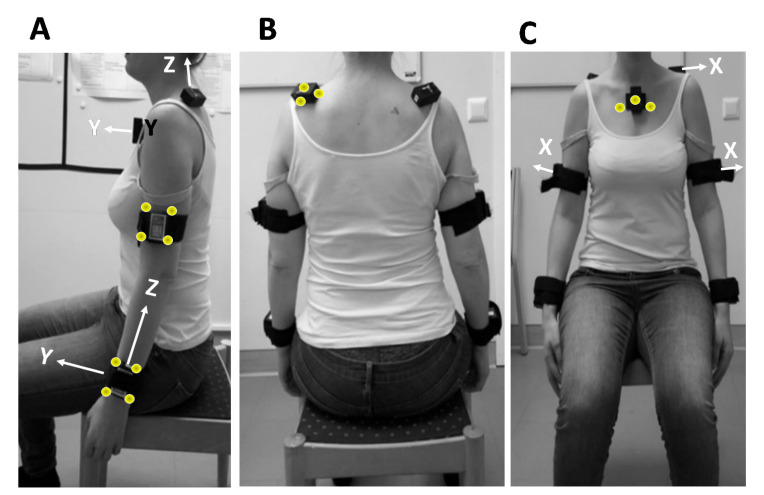
(**A**–**C**). Illustrates the IMU placements used in Part I (validity) and Part II (reliability). The reflective markers used in Part II were placed either on an orthoplastic shell with the sensor in the center of the shell (upper and lower arms, (**A**) or directly on the sensor’s front and sides (scapula (**B**) and thorax (**C**)). The local coordinate systems, marked with white arrows (**B**,**C**), were all defined so that after calibration/sensor alignment, they were oriented in the same way.

The IMUs were placed on upper arms, forearms, scapula, and thorax according to recommendations from our previous study [32]. The thorax sensor was placed on the chest, centered on the manubrium right under the jugular notch. The scapula sensor was placed cranially on the middle part of spina scapulae by palpating the spina scapulae from the most lateral part of acromion to the most medial part of the scapula. The upper arm sensor was placed at the distal part at a distance corresponding to one-third of the arm’s length. The forearm sensor was placed dorsally at the distal end close to the ulnar process. IMUs placed on the scapula and sternum were attached with double-sided surgical tape, and the sensors placed on the arms were fixed using elastic Velcro straps (Figure 1).

In Part I, orthoplastic shells with four reflective markers each were placed over the person’s forearm and upper arm, and the IMU was placed in the center of each shell on the anatomical positions specified above. On the thorax and scapula, the IMUs were attached first using double-sided surgical tape, and the reflective markers were then attached directly to the sensor. Data were sampled synchronously at 200 Hz (optical system) and 100 Hz (IMU system). An external trigger was used in order to start the systems synchronously.

### 2.4. Data Processing and Outcome Measure Calculations

The MoLab™ Analysis software (version 1.7, AnyMo AB, Umeå, Sweden) was used for sensor data preprocessing and analysis. First, the raw data from the gyroscope, accelerometer, and magnetometer were combined into joint angles through a fusion algorithm [33]. The fusion filter gain β (i.e., the parameter controlling by which amount the accelerometer and magnetometer data were used to correct the orientation estimated by the gyroscope) was set to 0.03.

For Part I (validity), the helical angle [34] was derived from the IMU system and the optical reference system, respectively, for each segment and task. In this part, we analyzed errors due to system differences only (not differences related to segment model differences between the IMU-based model compared to the marker-based model). Therefore, we calculated segment angles (the difference between marker cluster and sensor for each segment) instead of relative angles. The segment angles were based on a rigid cluster compared to the sensors which were centered in the middle of the cluster to ensure that the same position on each segment was analyzed. We also applied the helical angle instead of Cardan–Euler angles since this removes the effect of eventual misalignment of the system’s local coordinate axis when calculating the total error. Quaternion data from the IMU system were exported to Matlab (R2018b, Mathworks Inc., Natick, MA, USA) where the helical angular data were computed. Since we did not analyze how individual differences (such as weight, height, or joint function) affected the angle, we only collected data from a small sample of three persons. The data from the optical system were analyzed as follows: Missing marker data due to hidden markers were replaced using linear interpolation. The 3D positions of each marker cluster, four markers each, were used to compute direction cosine matrices (DCMs) based on a rigid body model [35]. Helical angular data were then computed from DCMs and resampled from 200 Hz to 100 Hz (i.e., the same frequency as the IMU system). The helical angular data from each system were filtered using a 5 Hz Butterworth antialiasing filter, and then the helical angular difference (IMU data compared to optical data) was computed for each segment and task.

For Part II, cardan angles were used for all analyses. For each segment, Y pointed in the segment’s anterior–posterior direction, Z pointed in the segment’s superior–inferior direction, and X in the segment’s mediolateral direction. The cardan sequence was chosen based on the movement plane where the movement mainly occurred to avoid gimbal lock [36]. For all other cases, the XYZ sequence was used according to the recommendations from the International Society of Biomechanics (ISB). The shoulder joint was defined by angular motion of humerus relative to thorax to describe flexion–extension (X), abduction–adduction (Y), and internal–external rotation (Z). The glenohumeral joint was defined by the motion of the humerus relative to the scapula to describe flexion–extension (X), abduction–adduction (Y), and internal–external rotation (Z). The scapulothoracic joint, defined by the motion of the scapula relative to the thorax, described anterior–posterior tilt (X), upward–downward rotation in the frontal plane (Y), and inward–outward rotation (protraction–retraction) in the horizontal plane (Z). The elbow joint was defined by the motion of the forearm relative to the upper arm to describe elbow flexion–extension (X) and forearm pronation–supination (Z).

The start (motion initiation) and stop (endpoint reached) events were set in the software program. All events were checked manually by visually scrutinizing shoulder and elbow joint angle curves and skeleton model animations for each task and trial to ensure that they corresponded to the correct point in time. Within each start and stop event, the following outcome measures were derived:

- Peak angles (maximum and minimum) in all joints and all three planes;
- Range of motion (RoM, peak maximum–peak minimum) in all joints and all three planes;
- Average angular speed in shoulder motion in all three planes, defined as shoulder RoM*mean performance frequency (°/s), where mean performance frequency is defined as number of repetitions/second.

A subset of outcome measures was selected according to their clinical relevance for each specific task. For example, the elbow mobility tasks were done to analyze secondary effects on the elbow joint, so scapula and shoulder motion was not analyzed in those tasks. MMS1 was graded according to the shoulder abduction range while MMS2 was graded according to shoulder rotation, hence outcome measures focused on the movement plane of interest for these two tasks. MMS3 was graded according to whether the neck was reached and graded clinically according to abduction range. Further, velocity measures were analyzed in the tasks MMS3 and MMS4 only since these were the two tasks where BPBI patients may use increased movement velocity to be able to fulfill the tasks. MMS5 and MMS6 involved mainly shoulder rotation but compensatory abduction could occur. This gave, in total, 69 outcome measures for statistical analyses.

### 2.5. Statistics

In Part I, the angular difference between the helical angle from the IMU system in comparison to the helical angle derived from the optical reference system and the reflective markers was computed for each segment and task. Bland–Altmann plots were used to identify systematic differences between the systems. The angular error was defined as the difference between the reference system’s angle and the IMU system angle, and the 95% confidence interval of this error was calculated as (−19.6 × SD, 1.96 × SD) where SD is the standard deviation of the angular error. For all errors, >10° was considered poor system accuracy, errors between 5 to 10° were considered moderate system accuracy, and <5° was considered high system accuracy. These limits were deemed as appropriate for kinematic analyses of BPPI patients since changing classification grade in the modified Mallet scale requires a difference of 20° in global external rotation and 30° in global abduction. Moreover, de Winter et al. chose 10° as the upper limit for an acceptable interrater difference when measuring shoulder range of motion with a digital inclinometer [37]. This was based on clinical experience, as no clear criteria for the acceptable degree of interobserver agreement were available [37].

In Part II, histograms were used to illustrate group averages and group standard error of measurements (SEM) for these selected outcome measures. First, each subject’s average was calculated from the test and retest session. Then, these subject average values were used to construct the group average and the group standard error of measurement. Group differences between measures from the BPBI-affected side and measures from the CTRL nondominant side were analyzed with paired *t*-tests. The alpha value was set to 0.05 for all statistical tests. To analyze test–retest and interrater reliability, the intraclass correlation coefficient (ICC) was calculated. For both test–retest and interrater reliability, an ICC value below 0.50 was considered as low reliability, ICC between 0.50 and 0.75 was considered moderate, ICC between 0.75 and 0.90 was defined as good reliability, and ICC > 0.90 as excellent reliability [15,38]. To analyze test–retest reliability, a mean value from the five trials from each occasion was used. The interrater reliability was analyzed for MMS2 global external rotation and MMS3 hand to neck and the BPBI group only. The ICC was computed based on the mean values from two test sessions done by the two independent test leaders.

## 3. Results

### 3.1. Validity

**Figure 2:**
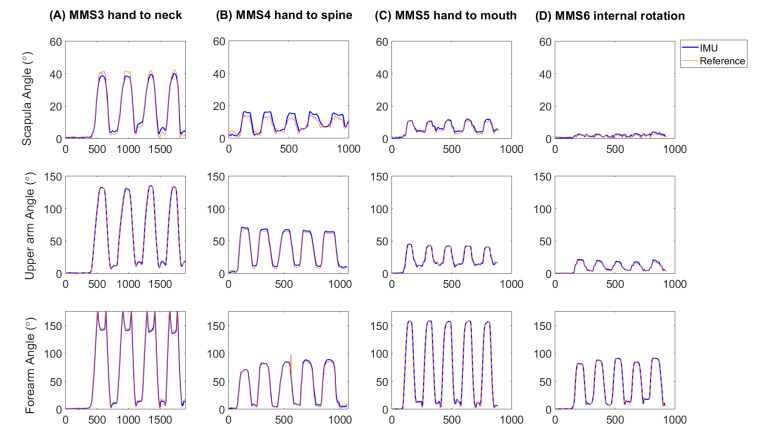
Examples of data from one test person during four of the tasks included in the modified Mallet score MMS3 hand to neck (**A**), MMS4 hand to spine (**B**), MMS5 hand to mouth (**C**), MMS6 internal rotation (**D**) as simultaneously measured with the reference system (red line) and the IMU system (thick blue line). The segment helical angle was calculated for scapula (upper row), upper arm (middle row) and forearm (bottom row).

**Figure 3:**
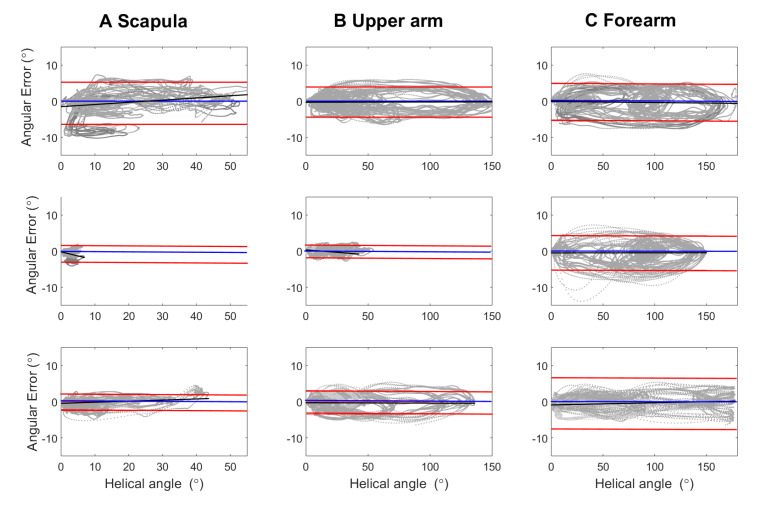
Bland–Altmann plots illustrate system agreements of the IMU system with the reference system. The helical angle was calculated for each segment, scapula (**A**), upper arm (**B**) and forearm (**C**). Angular errors were calculated for tasks involving large shoulder movements in one plane (upper row, shoulder flexion−extension, MMS1 global abduction and MMS2 global external rotation), for elbow mobility tasks (middle row, elbow flexion−extension and forearm pronation–supination); tasks involving movement in both elbow and shoulder (bottom row, MMS3 hand to neck, Mallet MMS4 hand to spine, MMS5 hand to mouth and MMS6 internal rotation). The mean error is illustrated with a blue line, and the error 95% confidence intervals are marked with red lines.

Examples of time series joint angle data from the simultaneous, synchronized measurements with the IMU system and the reference optical camera system are illustrated in Figure 2. Bland–Altmann plots showed that the IMU system had high accuracy, i.e., mean measurement errors between 0.2 to −0.7°, see Figure 3 and Table 2.

### 3.2. Reliability

To summarize, 90.3% of the outcome scores had good-to-excellent test–retest reliability. Further, 41% of the variables had both good-to-excellent test–retest reliability and also showed a significant group difference in the BPBI-affected arms versus control nondominant arms (*p* < 0.05). In general, outcome measures related to external–internal rotation in the shoulder and elbow had lower (i.e., moderate) reliability compared to other movement directions.

**Figure 4:**
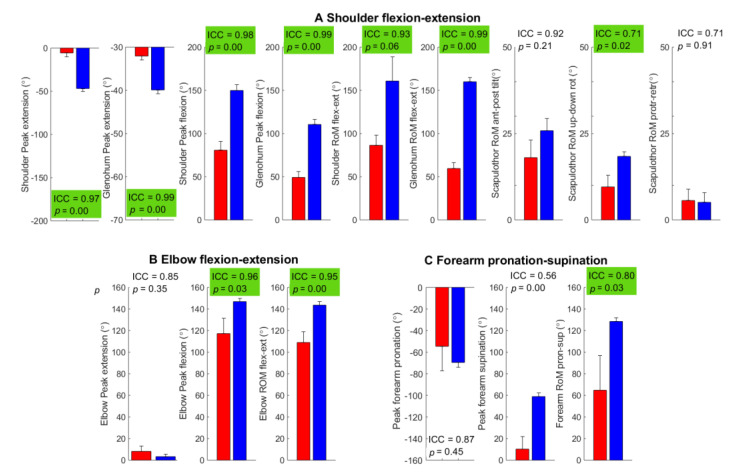
Outcome measures from the assessment of (**A**) shoulder and (**B**,**C**) elbow mobility. Group mean and standard error of mean are illustrated (BPBI: red left bar, control: blue right bar). ICC from test–retest reliability and *p*-values from *t*-tests are shown in the upper right corner of each subplot and are highlighted green for outcome scores with both good test–retest reliability (ICC > 0.75) and a significant group difference (*p* < 0.05).

The joint mobility tasks gave reliable peak and RoM outcome scores except in peak supination during forearm pronation–supination, which had moderate reliability (Figure 4). The BPBI patients had significantly reduced peak angles and RoM in the shoulder, glenohumeral, and elbow flexion–extension, with excellent repeatability (ICC 0.97–0.99). They also had repeatable, reduced RoM in the scapulothoracic upward–downward rotation compared to controls.

**Figure 5:**
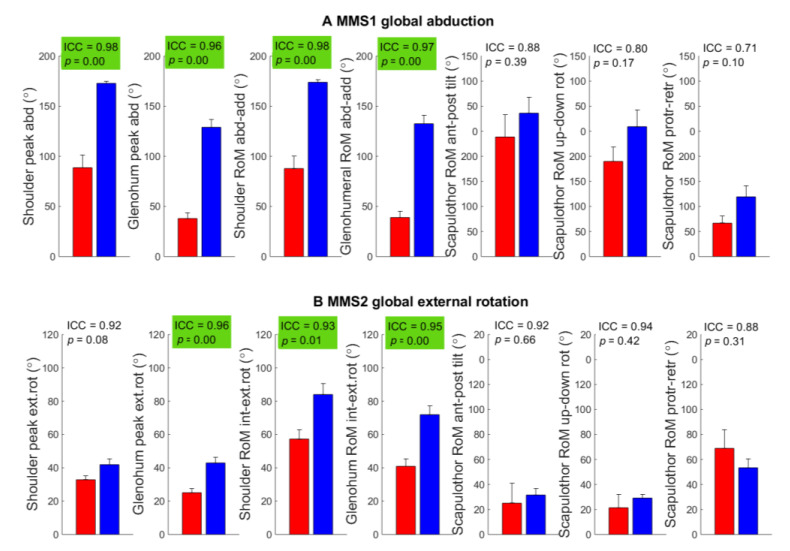
Outcome measures from the assessment of two tasks from the modified Mallet scale (MMS); (**A**) global abduction and (**B**) global external rotation. Group mean and standard error of mean are illustrated (BPBI: red left bar, control: blue right bar). ICC from test–retest reliability and p-values from *t*-tests are shown in the upper right corner of each subplot and are highlighted green for outcome scores with both good test–retest reliability (ICC > 0.75) and a significant group difference (*p* < 0.05).

**Figure 6:**
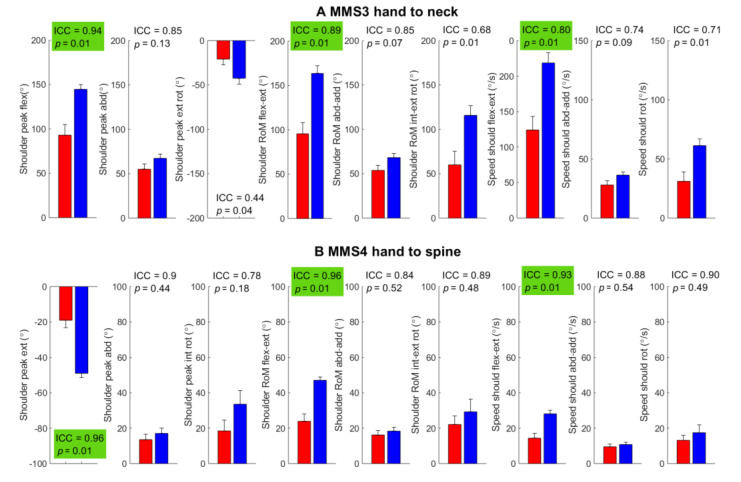
Outcome measures from the assessment of two tasks from the modified Mallet scale (MMS); (**A**) hand to neck and (**B**) hand to spine. Group mean and standard error of mean are illustrated (BPBI: red left bar, control: blue right bar). ICC from test–retest reliability and *p*-values from *t*-tests are shown in the upper right corner of each subplot and are highlighted green for outcome scores with both good test–retest reliability (ICC > 0.75) and a significant group difference (*p* < 0.05).

**Figure 7:**
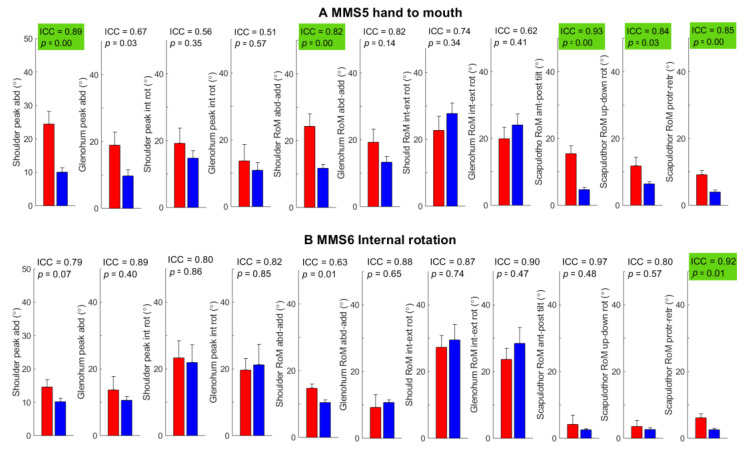
Outcome measures from the assessment of two tasks from the modified Mallet scale (MMS); (**A**) hand to mouth and (**B**) internal rotation. Group mean and standard error of mean are illustrated (BPBI: red left bar, control: blue right bar). ICC from test–retest reliability and p-values from *t*-tests are shown in the upper right corner of each subplot and are highlighted green for outcome scores with both good test–retest reliability (ICC > 0.75) and a significant group difference (*p* < 0.05).

Outcome measures from MMS1 (global shoulder abduction) and MMS2 (global external rotation) showed good-to-excellent test–retest reliability (ICC = 0.80–0.98) in all outcome measures except in scapulothoracic RoM which was moderate (ICC = 0.71) with significantly less RoM in the abduction and external rotation in the shoulder and glenohumeral joints in BPBI patients compared to controls (Figure 5). MMS3 hand to neck and MMS4 hand to spine showed good-to-excellent test–retest reliability in all outcome measures (ICC = 0.76–0.97) except in average angular speed in shoulder abduction–adduction (ICC = 0.73); BPBI patients showed a significantly reduced range in shoulder movements and a significantly lower average angular speed in shoulder flexion–extension compared to controls (Figure 6). MMS5 hand to mouth and MMS6 internal rotation also showed moderate-to-excellent test–retest reliability with significantly greater scapula motion in BPBI patients as compared to controls (Figure 7). The interrater reliability analysis for BPBI patients showed moderate-to-excellent reliability for all outcome measures during MMS2 global external rotation and MMS3 hand to neck (Table 3).

## 4. Discussion

In this study, we evaluated if the clinical test battery used in the assessment of brachial plexus injury could be improved if it was extended to include kinematic outcome scores from an IMU-based system. In summary, all outcome scores were reliable (ICC > 0.50), 88% (61 of 69) showed good-to-excellent test–retest reliability (ICC > 0.75, Figure 4, Figure 5, Figure 6 and Figure 7), and the validity was moderate-to-high for all tests and segments with mean error differences below 1°.

### 4.1. System Validity

The major limitations of marker- or IMU-based systems are that they are skin-based and affected by skin and tissue movement. Such error effects are especially large for joints covered with large muscle groups and soft tissues, such as the hip joint [39], and calculation of inward–outward rotation is commonly most affected both for the hip [39] and the arm [40] while flexion–extension angles are less affected. These limitations are important to be aware of when implementing these methods in clinic, for example, by focusing on movement tasks and movement directions that are least affected by soft tissue artifacts. Despite these limitations, the optical marker-based motion capture system is considered the gold standard method in human motion analysis, especially for clinical purposes since it is noninvasive and has a high measurement accuracy. In the current study, we analyzed the IMU system’s validity in comparison with an optical system based on segment helical angles. For all segments and tasks, the error mean was low (below 1°) with acceptable 95% confidence intervals (CI). The largest 95% CI was found for the lower arm segment during tasks involving large shoulder and elbow movements (i.e., when the subject tried to reach the back, mouth, or neck) with a linear slope of 0.04. This could imply a sensitivity drift during large movements that gives a small systematic error in the IMU compared to the reference system.

### 4.2. Reliability of Shoulder, Scapula, and Elbow Movement

Peak internal and external rotation of the shoulder and forearm appeared to be the least reliable; for example, the interrater reliability of shoulder rotation was moderate (ICC 0.55–0.62), and the test–retest reliability for shoulder and glenohumeral internal rotation was moderate during MMS5 hand to mouth (ICC 0.51–0.56). This could relate to the fact that rotations around a segment’s long axis tend to be difficult to measure reliably [40,41,42]. A possible explanation is that a relatively large outward rotation in the upper arm causes a rather small movement of the sensor compared to possible artifacts from the upper arm’s musculature and/or skin. That is, the shoulder joint rotates substantially under the skin without the sensor following along which leads to a larger error compared to the error that arises during flexion/abduction of the upper arm where the sensor smoothly follows the movement. Moreover, due to anatomical differences and contractures, BPBI patients tend to have less control over rotational movement [2,15], something that may have influenced the reliability of the outcome scores from the BPBI group.

The scapula segment is challenging to measure with IMU sensors since movement magnitudes are small and may be hidden by overlying tissues [23]. Scapula movement also involves linear displacements in the superior–anterior direction (such as during elevation and depression) and in the anterolateral and posteromedial directions (during protraction–retraction) [41] which cannot be measured directly by IMUs. Even so, several tasks gave clinically relevant information about scapula movements (i.e., outcome scores with ICC > 0.75 and with significant group differences), such as significantly increased RoM in scapula motion in BPBI patients during MMS5 hand to mouth and MMS6 internal rotation. This is in agreement with the studies by Russo et al., who also found an increased scapulothoracic motion in BPBI patients during this task [43], and Duff et al., who found that the scapulothoracic joint had a greater contribution to arm elevation in BPBI patients [44]. The validity of the scapula segment was also high in comparison to the gold-standard optical system for these tasks (−0.2 ± 1.2°; MMS3-6). Hence, we conclude that scapula RoM indeed can be estimated by IMU-based systems even though the magnitude of motion must be interpreted with caution due to soft tissue artifacts.

In clinical assessment, performance speed can be visually observed during the Mallet hand to neck and hand to spine tasks to see if the BPBI patient used speed to compensate for restricted arm and shoulder function. In the current study, average speed outcome measures were derived and had moderate-to-excellent test–retest reliability (ICC = 0.71–0.93). The average speed was lower in BPBI patients compared to controls. One reason for this was that this task was standardized so that the patients were encouraged to perform the task in a controlled way. Future studies could investigate whether peak speed could be analyzed instead of average speed to get information about movement control.

Elbow mobility was examined since secondary complications, such as elbow contractures, are common after a plexus brachialis injury. This is relevant to follow-up and habilitate because when left untreated, these can lead to functional and aesthetic problems [5]. The elbow mobility tests revealed significantly decreased RoM in elbow flexion–extension and pronation–supination in BPBI patients compared to controls with good to excellent reliability. Forearm supination had moderate reliability (ICC = 0.56) even though it revealed a significant reduction in supination in BPBI patients. As mentioned above, the internal rotation of a joint is in general difficult to measure reliably with the current method. Hence, RoM in pronation–supination (ICC = 0.80, *p* = 0.03) is preferable compared to peak values for clinical purposes.

The interrater reliability was moderate to excellent for all selected outcome measures. Some differences in interrater versus test–retest reliability were observed, for example, in shoulder external rotation which had excellent test–retest reliability in MMS3 hand to neck (ICC = 0.97) but moderate interrater reliability (ICC = 0.62). This could imply that the instructions to the patients differed slightly between the two test leaders, highlighting that standardized instructions and procedures become very important when patients are followed-up with over a long period of time. A limitation of the current study was that the interrater reliability was only analyzed for two of the tasks. The reasons were (1) to avoid fatigue for the BPBI patients since performing the complete test battery twice would be demanding for these patients, which would have negatively affected the repeatability and (2) because the measurements were performed by clinical staff during an ordinary clinical appointment, which limited the amount of available time for each evaluation. Further studies should be designed so that a full evaluation of the complete modified Mallet scale is enabled.

### 4.3. Methodological Aspects and Clinical Implications

The modified Mallet scale is widely used in BPBI assessment [5,12,13], and combining this instrument with IMU sensors could partly eliminate known problems with the instrument’s limited objectivity and sensitivity. This study showed that the IMU system indeed gave relevant and reliable clinical information about shoulder function. This also enabled analyses of scapular movement and speed, which are important aspects that cannot be graded either with the modified Mallet scale [11] or goniometer/eyeballing [45], which are also commonly used in clinic.

The implementation of the system into the clinical test battery was rather straightforward, and the clinicians in the current study could perform the measurements and analyses after a few training sessions. The movement registrations did not take more than about 30 min if preparations (such as calibration and setting up the segment model) were made beforehand. In the current study, IMUs with gyroscopes, accelerometers, and magnetometers were utilized. Great caution was taken to ensure that no magnetic disturbances compromised the measurements. A practical problem is that most hospitals have magnetic materials in walls, floors, and surrounding equipment that give magnetic disturbances. In that case, magnetometers can be excluded, and a simplified calibration procedure needs to be utilized, assuming that all joint angles are zero during the standardized starting position [46]. In this case, we recommend RoM values to avoid systematic errors related to BPBI patients having difficulties holding their arms in such a straight, standardized position.

The current study was based on a small sample and should be extended to larger patient groups, and the results should be related to clinical findings and rehabilitation progress. It would be a great benefit to evaluate if IMU-based methods are suitable for younger children since early assessments are very important but also the most difficult to perform.

## 5. Conclusions

Inertial sensors could detect shoulder motion with high validity and good-to-excellent test–retest reliability and interrater reliability. Internal–external rotation appeared to be the least reliable, and caution should be taken when analyzing motion in this plane. The instructions from the clinician to the patient are also important if the patient is followed-up longitudinally. The inclusion of inertial sensors in a clinical test battery used to assess shoulder function could improve the assessment of patients with brachial plexus injury.

## Figures and Tables

**Table 1:** Description of participants for Part II of this study (Test–retest and interrater reliability).

Subject Data	BPBI	Control
Sex (F/M)	4 F, 2 M	6 F, 3 M
Age (Years)	16.8 (8–22)	19.7 (8–25)
Affected side	2 R, 4 L	N/A
Preferred side	Non-affected ^1^	2 L, 7 R
Day between examinations	5.8 (2–8)	4.1 (2–7)

^1^ In all cases, the nonaffected side was also the preferred side.

**Table 2:** Comparison between a reference system and the IMU system. The mean error (MR), the error confidence interval (95% CI), and error slope (k) from linear regression are presented for different types of movements. The error is based on the segment helical angle.

Statistics	Task Type	Scapula	Upper Arm	Lower Arm
ME (95% CI)	Shoulder one plane ^1^	−0.6 (−6.4, 5.2)	−0.3 (−4.4, 3.9)	−0.3 (−5.4, 4.8)
	Elbow one plane ^2^	−0.6 (−2.9, 1.7)	0.2 (−1.6, 1.9)	−0.7 (−5.4, 4.1)
	Shoulder and elbow ^3^	−0.3 (−2.6, 2.1)	−0.3 (−3.5, 2.8)	−0.6 (−7.5, 6.4)
k	Shoulder one plane ^1^	0.040	0.000	0.004
	Elbow one plane ^2^	−0.432	0.000	−0.001
	Shoulder and elbow ^3^	0.021	−0.002	0.004

^1^ Shoulder flexion–extension, MMS1 global abduction, and MMS2 global external rotation ^2^ Elbow flexion–extension, and elbow supination–pronation ^3^ MMS3 hand to neck, Mallet MMS4 hand to spine, MMS5 hand to mouth, MMS6 internal rotation.

**Table 3:** Interrater reliability for two test leaders assessing BPBI patients in MMS2 global external rotation and MMS3 hand to neck.

Task	Joint	Outcome Score	ICC (*p*-Value)
MMS2	Shoulder	Maximal ext rotation	0.55 (0.22)
		RoM IE	0.62 (0.18)
	Glenohumeral	Maximal ext rotation	0.71 (0.09)
		RoM IE	0.90 (0.00)
	Scapulothoracic	RoM ant–post	0.93 (0.00)
		RoM up–down	0.82 (0.04)
		RoM protr–retr	0.90 (0.00)
MMS3	Shoulder	Peak flexion	0.89 (0.02)
		Peak abduction	0.76 (0.05)
		Peak external rotation	0.62 (0.11)
		RoM FE	0.91 (0.01)
		RoM ab–add	0.83 (0.03)
		RoM IE	0.81 (0.04)
		Average angular speed FE	0.82 (0.03)
		Average ang speed ab–add	0.72 (0.07)
		Average ang speed IE	0.69 (0.08)

## Data Availability

The data presented in this study are available on request from the corresponding author.

## Supplementary Materials

The following supporting information can be downloaded at: https://www.mdpi.com/article/10.3390/s22239557/s1, Figure S1: Description of motion tasks used in the study.
