# Supplementary material for: Validity and Reliability of Wearable Motion Sensors for Clinical Assessment of Shoulder Function in Brachial Plexus Birth Injury
Source: Sensors (Basel). 2022 Dec 6;22(23):9557. doi: 10.3390/s22239557 (PMC9736020; doi:10.3390/s22239557)
Supplement: Supplementary file 1 [file sensors-22-09557-s001.zip › sensors-2013110-supplementary.pdf]

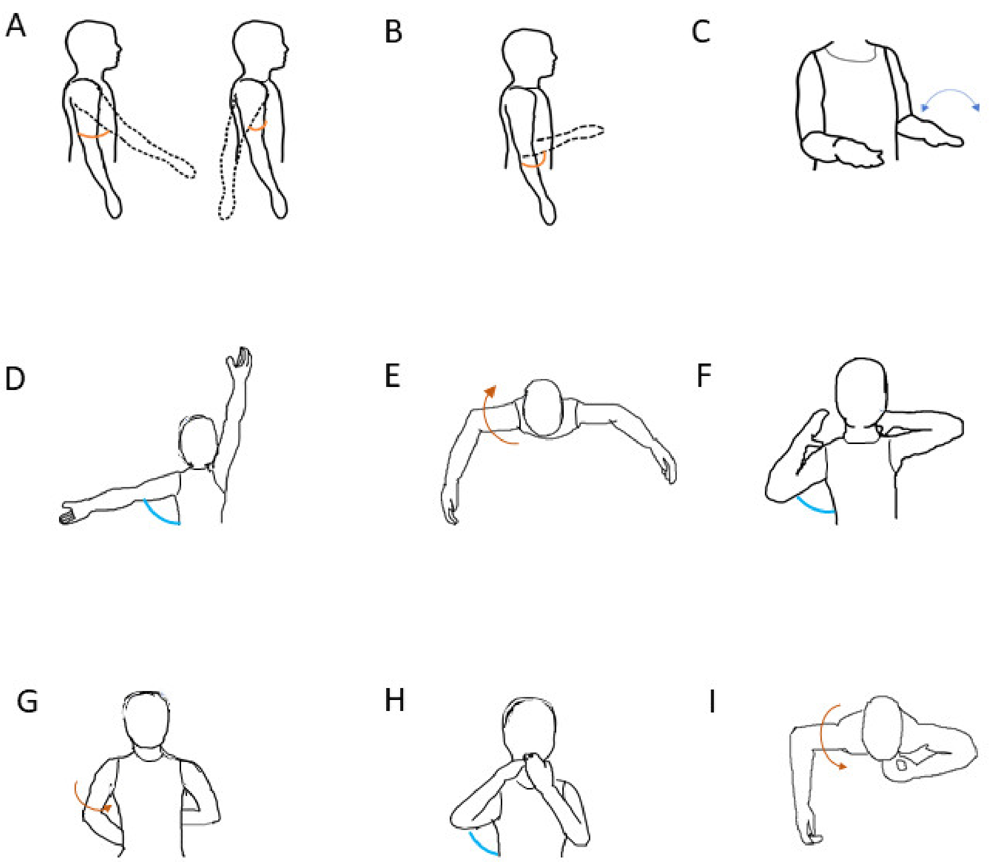

Figure S1: Description of motion tasks used in the study. (**A**–**C**) summarizes the three motion tasks used to investigate joint motion range, all commonly measured with goniometer in clinic: shoulder flexion–extension (**A**), elbow flexion–extension (**B**), and forearm pronation–supination (**C**). Figure (**D**–**I**) describes the six motion tasks based on the modified Mallet scale, see [11] for details: (**D**) global abduction (range in global abduction is graded visually), (**E**) global external rotation (range in external rotation is graded visually), (**F**) hand to neck (the ability to reach the neck is graded in clinic), (**G**) hand to spine (the ability to reach the back is graded in clinic), (**H**) hand to mouth (the ability to reach the mouth without abduction is graded in clinic) and (**I**) internal rotation (the ability to reach the belly without wrist flexion is graded in clinic). Motion directions and angles of clinical interest when grading dysfunction are marked in each figure (**D**–I), where the person's right arm represents the injured side.
